# Combinations of EGFR and MET inhibitors reduce proliferation and invasiveness of mucosal melanoma cells

**DOI:** 10.1111/jcmm.17935

**Published:** 2023-09-07

**Authors:** Aleksandra Simiczyjew, Justyna Wądzyńska, Magdalena Kot, Marcin Ziętek, Rafał Matkowski, Mai P. Hoang, Piotr Donizy, Dorota Nowak

**Affiliations:** ^1^ Department of Cell Pathology, Faculty of Biotechnology University of Wroclaw Wroclaw Poland; ^2^ Department of Oncology and Division of Surgical Oncology Wroclaw Medical University Wroclaw Poland; ^3^ Lower Silesian Oncology Pulmonology and Hematology Center Wroclaw Poland; ^4^ Department of Pathology Massachusetts General Hospital, Harvard Medical School Boston Massachusetts USA; ^5^ Department of Clinical and Experimental Pathology Wroclaw Medical University Wroclaw Poland; ^6^ Department of Pathology and Clinical Cytology Jan Mikulicz‐Radecki University Hospital Wroclaw Poland

**Keywords:** crizotinib, EGFR, foretinib, lapatinib, MET, mucosal melanoma, signalling pathways

## Abstract

Mucosal melanoma (MM) is a very rare and aggressive type of cancer for which immunotherapy or targeted therapy such as BRAF/MEK inhibitors, used in cutaneous melanoma, usually fail. Due to our earlier experience showing the high effectiveness of epidermal growth factor receptor (EGFR) and hepatocyte growth factor receptor (MET) inhibitors in reducing the activation of the MAPK and PI3K/AKT signalling pathways, we aim to test whether these drugs would also be effective for mucosal melanoma. Cells representing two commercially available mucosal melanoma cell lines (GAK and HMVII) and one cell line obtained from a patient's vaginal melanoma were treated with MET or EGFR inhibitors, or combinations of these agents. The dual‐inhibitor treatment strategy resulted in a decrease of cell proliferation, migration and invasion. Moreover, combinations of inhibitors led to reduction of pEGFR/EGFR and pMET/MET ratio and downregulation of PI3K/AKT and MEK/ERK1/2‐based signalling pathways. Our findings indicate a potential therapeutic strategy based on EGFR and MET inhibitors in mucosal melanoma, which should be further evaluated in vivo and in clinical experiments. They also suggest that targeting multiple receptor tyrosine kinases may block signalling crosstalk and possibly delay the appearance of resistance to kinase inhibitors in mucosal melanoma cells.

## 1 INTRODUCTION

Mucosal melanoma (MM) is a rare disease, differing in many ways from cutaneous melanoma (CM). The role of melanocytes present in the mucosal membranes is not well known, but it may be related to antimicrobial and immunologic functions.^1^, ^2^, ^3^, ^4^ MM represents approximately 1.5% of all melanoma cases and most often affects the sinonasal tract (55.4%), the anus/rectum (23.8%) and the female genital tracts (18%).^5^, ^6^, ^7^ Due to the invisible sites of occurrence, MM is usually diagnosed at advanced stage. Moreover, this neoplasm is characterized by the ability to form early metastasis. The above‐mentioned facts explain the overall poor prognosis of patients with MM.^7^, ^8^ For CM, the 5‐year survival rate is 80.8%, while for MM it is only 25%.^3^, ^9^ Due to the rarity of the disease, large‐scale clinical trials have not been conducted and epidemiological data remain incomplete.

For MM, surgical resection is typically the treatment of choice yet their anatomic locations prevent wide excision in order to obtain negative surgical margins. Therefore, it is not surprising that 50%–90% of patients experience local recurrence.^3^, ^4^, ^8^, ^10^ Immunotherapy in a form of anti‐CTLA‐4 and anti‐PD‐1/PD‐L‐1 antibodies appears to be more effective than previously used classical chemotherapy based on cisplatin, vinblastine and dacarbazine, although in much less extent in MM than in CM.^3^, ^4^, ^11^, ^12^, ^13^ Furthermore, current targeted therapeutic options available for MM patients are very limited. Mucosal melanoma shows increased genomic instability and lower rates of somatic mutations in comparison with CM. Frequently mutated genes in MM are as follows: *NRAS* (14%–30%), *BRAF* (5%–16%), *NF1* (16%), *KIT* (5%–15%), *SF3B1* (12%), *TP53* (8.9%) and *SPRED1* (7%).^14^ Mutations in *BRAF* and *NRAS* observed in MM are substantially different from those occurring in CM.^4^, ^7^, ^15^ *BRAF* mutation was present in 26% of patients with vulvar and vaginal melanoma; however, only half of them were V600 mutations, which significantly reduces the effectiveness of BRAF and MEK inhibitors therapy typically used in CM.^16^ For the minority of metastatic MM patients who exhibit BRAF V600E mutations, combined anti‐BRAF and anti‐MEK therapy may represent an adequate therapeutic strategy.^17^, ^18^ Activating *KIT* mutation or amplification would be a promising therapeutic target in MM, but patients treated with KIT inhibitors acquire drug resistance shortly after treatment leading to progression of the disease. *NF1* is one of the most often altered genes in MM (less than 20%), mainly through mutations leading to a loss of function. This protein inactivation results in hyperactivation of MAPK pathway.^7^, ^19^, ^20^ Moreover, for patients with *NRAS*, *NF1* or *SPRED1* alterations, a potential therapeutic option may be targeting the downstream protein MEK. Unfortunately, the acquired resistance through the reactivation of the MAPK pathway may occur in patients treated with both the MEK inhibitor used alone and in combination with BRAF inhibitor.^17^ Moreover, the PI3K/AKT signalling cascade is also frequently activated in MM. This activation occurs, similar as in the case of MAPK pathway, as a result of mutations in *BRAF*, *KIT*, *NRAS*, *NF1* and *SPRED1*.^1^, ^21^ Blocking of MEK and mTORC1/2 at the same time, using trametinib and sapanisertib leads to apoptosis and reduction of canine MM cells survival, although these results have not been confirmed on human MMs.^18^ The development of new treatment strategies, based on immunotherapy or small molecule inhibitors, significantly improved the prognosis of CM patients, but did not substantially affect the prognosis of MM patients.^22^ As a result, MM patients suffer from limited treatment options and inadequate response. Above‐mentioned data indicate over‐activation of the MAPK and PI3K/AKT cascades and suggest that downregulation of these pathways could be an efficient form of therapy in MM.

Due to our earlier observation^23^, ^24^ that epidermal growth factor receptor (EGFR) and hepatocyte growth factor receptor (MET) inhibitors reduce the activation of the MAPK and PI3K/AKT signalling pathways, and thus significantly decrease the viability and invasion of CM, we decided to test whether these drugs would also be effective in MM. Our assumption about the potential efficacy of this drugs' combination was reinforced by the fact that Forschner et al. indicated that *EGFR* amplification occur in around 30% of MM patients, while Gottesdiener detected—belonging to EGRF family—*ERBB2* amplifications in 6% of MM.^25^, ^26^ Moreover kinase profiling conducted in a panel of melanoma cell strains showed that *EGFR* is often activated in melanoma cells.^27^, ^28^ Song et al. showed also that MM expresses comparable level of MET as that of primary and metastatic CM, much higher than in benign nevi (Song et al., 2020). MET inhibitors are often not effective as single‐therapy agents in melanoma in clinical studies, which may be related to the interaction of the MET based pathways with other oncogenic pathways. One of these interplays is MET–EGFR crosstalk, which was reported to be involved in therapeutic resistance to EGFR inhibitors in colon and lung cancers.^29^, ^30^ We hypothesize that in contrast to anti‐CM therapies which have often proven to be ineffective in the case of MM, a combination of MET and EGFR inhibitors may be effective in this instance.

## 2 MATERIALS AND METHODS

### 2.1 Cell culture

Two commercially available MM cell lines were used. GAK cells (RRID:CVCL_1225) representing vulvar melanoma were obtained from TebuBio and were grown in HAM's F12 (Sigma Aldrich) medium supplemented with 20% foetal bovine serum (FBS) (Gibco). HMVII cell line (RRID:CVCL_1282), representing vaginal melanoma, was purchased from Sigma Aldrich and grown in Ham's F10 medium (Sigma Aldrich) containing 10% FBS (Gibco). Both cell lines were purchased 2 years ago and were authenticated by the selling company. To both types of cell culture media were added 2 mM glutamine (Gibco), and antibiotic‐antimycotic solution (100 U/mL penicillin, 100 μg/mL streptomycin and 0.25 μg/mL Amphotericin B) (Gibco). The cells were cultured in 25 cm^2^ tissue culture flasks at 37°C in 5% CO^2^/95% humidified air and passaged once a week using a 0.25% trypsin/0.05% EDTA solution (IITD PAN Wroclaw). All experiments were performed with mycoplasma‐free cells.

### 2.2 Isolation of melanoma cells from patient's tumour

Mucosal melanoma sample representing primary tumour was obtained during surgical intervention in Lower Silesian Oncology, Pulmonology and Hematology Center, Wroclaw, Poland. Histopathological analysis was carried out to confirm the melanocytic characteristics of tumour specimen. A 47‐year‐old woman was diagnosed with vaginal melanoma categorized as pT4aN0M0. The study was permitted on the 2.06.2020 by the Ethical Committee of the Wroclaw Medical University, Poland (decision number: 337/2020). The experiments were executed with the understanding and written consent of patient involved in the study. The study methodologies conformed to the standards set by the Declaration of Helsinki. Melanoma cells were isolated as previously described by Sztiller‐Sikorska et al.^31^ Briefly, tumour fragments were minced using sterile scalpels and then incubated in RPMI 1640 medium (Sigma) supplemented with 0.01% DNase I (Sigma) and 0.5% collagenase IV (Invitrogen) for 2–3 h at 37°C. After centrifugation, isolated cells were seeded in complete medium (RPMI 1640 with 20% FBS) to the well of the six‐well Primaria plate (Corning). Twenty‐four hours later, the medium was changed to serum‐free medium consisting of DMEM/F12 (Gibco), B‐27 supplement (Gibco), 10 ng/mL basic fibroblast growth factor (PreproTech), insulin (10 mg/mL) (Sigma), heparin (1 ng/mL) (Sigma), EGF (20 ng/mL) and antibiotics (100 U/mL penicillin, 100 μg/mL streptomycin, 25 μg/mL amphotericin B). In order to subculture obtained cells, they were trypsinized using 0.25% trypsin/0.05% EDTA solution and then blocked with RPMI 1640 medium containing 10% FBS, centrifuged and seeded in the same medium. After 24 h, medium was changes for DMEM/F12 supplemented with above‐mentioned components. The obtained cell line was treated with selected inhibitors and analysed using immunocytochemical staining, proliferation tests, migration and invasion assays as well as analysis of the level of selected proteins by Western blotting.

### 2.3 Treatment of cells with inhibitors

The cells were incubated with diverse concentrations of EGFR inhibitor lapatinib (Santa Cruz Biotechnology, CAS 231277–92‐2), MET inhibitor crizotinib (Santa Cruz Biotechnology, sc‐3,564,471) or foretinib (Santa Cruz Biotechnology, CAS 849217–64‐7) separately or in combinations (crizotinib/lapatinib and foretinib/lapatinib) for 24 or 48 h. The inhibitors' concentrations used in the experiments (2 or 5 μM crizotinib, 2 or 5 μM foretinib and 5 or 7.5 μM lapatinib) were selected based on previous XTT experiments (data not shown). For each assay, cells were stimulated with 5 nM EGF (Corning) and 30 ng/mL HGF (Sigma Aldrich) to imitate conditions present in the melanoma microenvironment. Cells incubated only with growth factors and 0.1% DMSO (inhibitors solvent) were treated as a control.

### 2.4 Cell cytotoxicity assay

The Cell Proliferation Kit II (XTT) (Roche) was used according to the manufacturer's protocol. The XTT labelling mixture was added in parallel samples at time 0 (t0) and after 24 h (t24) or 48 h (t48) of cell growth in the absence or presence of inhibitors (crizotinib, foretinib, lapatinib or combinations) at the indicated concentrations. Absorbance was measured 3 h after XTT addition. All conditions were prepared in four replicates. Proliferation was calculated as the ratio of t48 or t24 to t0 in relation to the control, for which the value of 1 was assumed. Next, in the case of cell lines synergy scores for examined pairs of inhibitors were calculated using SynergyFinder+ software.^32^ It is considered that if the Loewe score is less than −10, the drug combination has an antagonistic effect, if the score is between −10 and +10, the drug combination has an additive effect, and if the score is greater than 10, the drug combination has a synergistic effect.

### 2.5 Cell migration and invasion

Cells were seeded into ImageLock 96‐well plates (IncuCyte) coated with Matrigel (BD Biosciences) with a concentration of 1 mg/mL and incubated for 24 h. Then, standardized wounds were generated in all wells simultaneously using Wound Maker™ (Essen Bioscience). To evaluate invasion, the cell‐free zone and the cells were covered with an additional Matrigel (1 mg/mL) layer. Then, cell culture medium containing growth factors and inhibitors was added on the cell layer directly (migration assay) or on the top of Matrigel matrices (invasion assay). Phase‐contrast time‐lapse images were captured with a time interval of 2 h, utilizing a 10 × objective in an IncuCyte® Live‐Cell Analysis System. Cells were allowed to cover the wound for 24 or 48 h. Representative results were further analysed using IncuCyte® Scratch Wound Cell Migration Software Module (Sartorius). The relative wound density represents the increase in the area covered by the cells over time in comparison with the control. Three repetitions of the experiments, each condition consisting of three replicates, were performed.

### 2.6 Western blot analysis

Cells were transferred on ice, washed three times with PBS and then lysed in urea‐containing buffer (50 mM Tris, pH 7.4, 5% SDS, 8.6% sucrose, 74 mM urea, 1 mM dichlorodiphenyltrichloroethane) supplemented with protease and phosphatase inhibitor cocktails (Sigma Aldrich). The protein concentration of the cell lysates was determined by a BCA procedure. Additionally, 24 h after seeding, cells were treated for 24 h with crizotinib, foretinib, lapatinib or their combinations in medium containing 30 ng/mL HGF and 5 nM EGF. Samples containing 10 μg of protein were analysed by SDS‐PAGE, according to the procedure described by Laemmli,^33^ and transferred to nitrocellulose sheets, according to Towbin et al.^34^ Membranes were blocked with 5% milk in TBST for 1 h. Primary antibodies: anti‐MET (SantaCruz Biotechnology, sc‐10, 1:200), anti‐p‐MET (Sigma, C7240, 1:500), anti‐EGFR (Cell Signalling, #4267, 1:1000), anti‐pEGFR (Genetex, 122,810, 1:500), anti‐AKT (Cell Signalling, #4691, 1:1000), anti‐pAKT (Cell Signalling, #9271, 1:1000), anti‐ERK1/2 (Cell Signalling, sc‐9102, 1:1000) and anti‐pERK1/2 (Cell Signalling, sc‐9101, 1:1000) were used. Goat anti‐mouse (Cell Signalling, 70,763, 1:4000) or goat anti‐rabbit (Cell Signalling, 70,743, 1:4000) antibodies conjugated to horseradish peroxidase (HRP) were applied according to the manufacturer's protocols. Immunoblots were developed using the Clarity Western ECL Substrate (Bio‐rad, Hercules, CA, USA) and scanned using the ChemiDoc gel scanner (Bio‐Rad). Quantitative analysis was performed using Image Lab software (Bio‐Rad). Results were normalized to total protein content assessed by Ponceau S staining. Protein phosphorylation rate was estimated by dividing the signal for phosphorylated protein by the signal for total protein that include phosphorylated and unphosphorylated form. All experiments were performed in three biological repetitions.

### 2.7 Immunocytochemistry

The subcellular distribution of actin filaments, cell nuclei, melanoma marker Melan A^35^ and MAGE‐C2 (marker of cancer cells)^36^ was examined by immunofluorescence. Cells were seeded on coverslips, and after 24 h, they were fixed with 4% formaldehyde and permeabilized with 0.1% Triton X‐100 in PBS. Coverslips were then blocked with 1% bovine serum albumin. Anti‐Melan A (Sigma, M6570) or anti‐MAGE‐C2 (Sigma, HPA062230) antibodies, followed by Alexa Fluor 488–conjugated secondary antibodies (anti‐mouse Invitrogen, A21202 or anti‐rabbit, Invitrogen, A21206), were applied to visualize these proteins. Actin filaments were stained with Alexa Fluor 568–labelled phalloidin and cell nuclei with Hoechst 33342. Then, coverslips were mounted with Dako fluorescent mounting medium. For each condition, representative cells are shown. Three repetitions of the experiments were performed.

### 2.8 Statistical analysis

All data are given as means +/− standard deviation (SD). Their significance was determined with GraphPad Prism 7 Software using one‐way anova followed by the Tukey test (i.e. cytotoxicity assay, migration and invasion assays, densitometric analysis of Western blots).

## 3 RESULTS

### 3.1 The combination of EGFR and MET inhibitors exerts cytotoxic effect on mucosal melanoma cell lines

**FIGURE 1:**
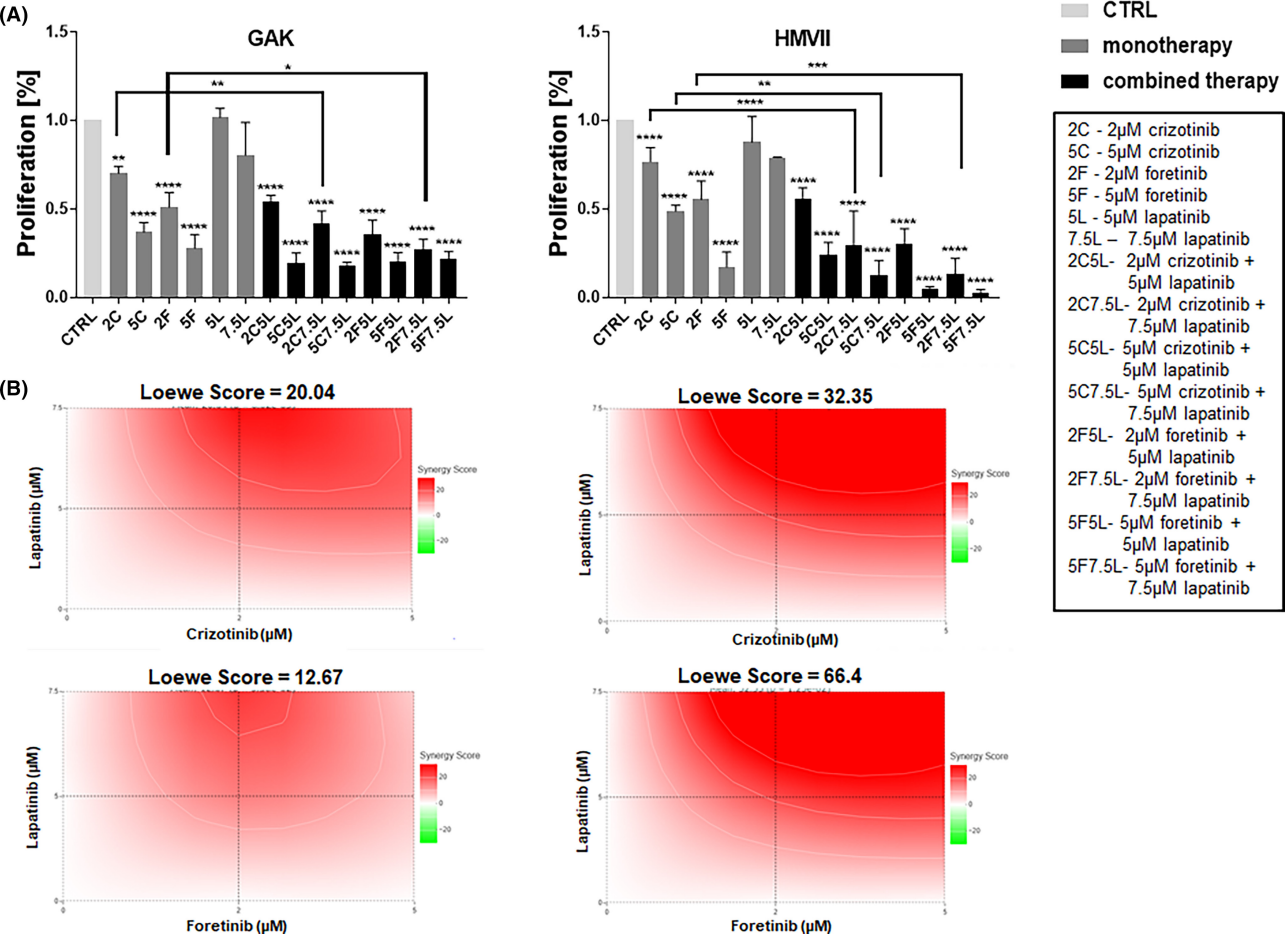
The influence of crizotinib (C), foretinib (F), lapatinib (L) and their combinations on mucosal melanoma cell lines proliferation. (A) Proliferation of GAK and HMVII cells after 48 h of incubation with the indicated concentrations of inhibitors (in μM) were compared to those of control cells (number of proliferating cells versus control with a control set up to 1). Results expressed as the mean ± SD are based on at least three independent experiments. The statistical significance was assessed versus the control and between particular conditions. The significance level was set at *p* ≤ 0.05 (*), *p* ≤ 0.01 (**), *p* ≤ 0.001 (***) or *p* ≤ 0.0001 (****). (B) Evaluation of synergy of examined drugs' combination. Analysis was performed in SynergyFinder software.

We first evaluated the cytotoxic effect of the single agents and their combinations using an XTT assay (Figure 1A). The cells were treated with lapatinib, crizotinib, foretinib or the combinations of them for 24 h in the presence of EGF and HGF. The applied inhibitors concentrations were selected based on toxicity tests using a broad concentration range (data not shown). The stimulation with both growth factors ensures that both signalling pathways initiated from EGFR and MET receptors are activated, which mimics physiological conditions in the tumour microenvironment. Crizotinib and foretinib reduced the percentage of proliferating cells similar as combinations of EGFR and MET receptors inhibitors. The effect of combination of crizotinib or foretinib and lapatinib was stronger than the effect exerted by crizotinib or foretinib used alone in both examined cell lines. Interestingly, both cell lines showed relative resistance to lapatinib as a single agent. We performed also an analysis of synergy of drugs combinations using SynergyFinder+ software. We observed that Loewe synergy score for both pairs: crizotinib/lapatinib and foretinib/lapatinib is higher than 10 for both cell lines, indicating that these combinations synergistically reduce the proliferation of the tested cells (Figure 1B).

### 3.2 Effect of inhibitors on the migration and invasion abilities of mucosal melanoma cells

**FIGURE 2:**
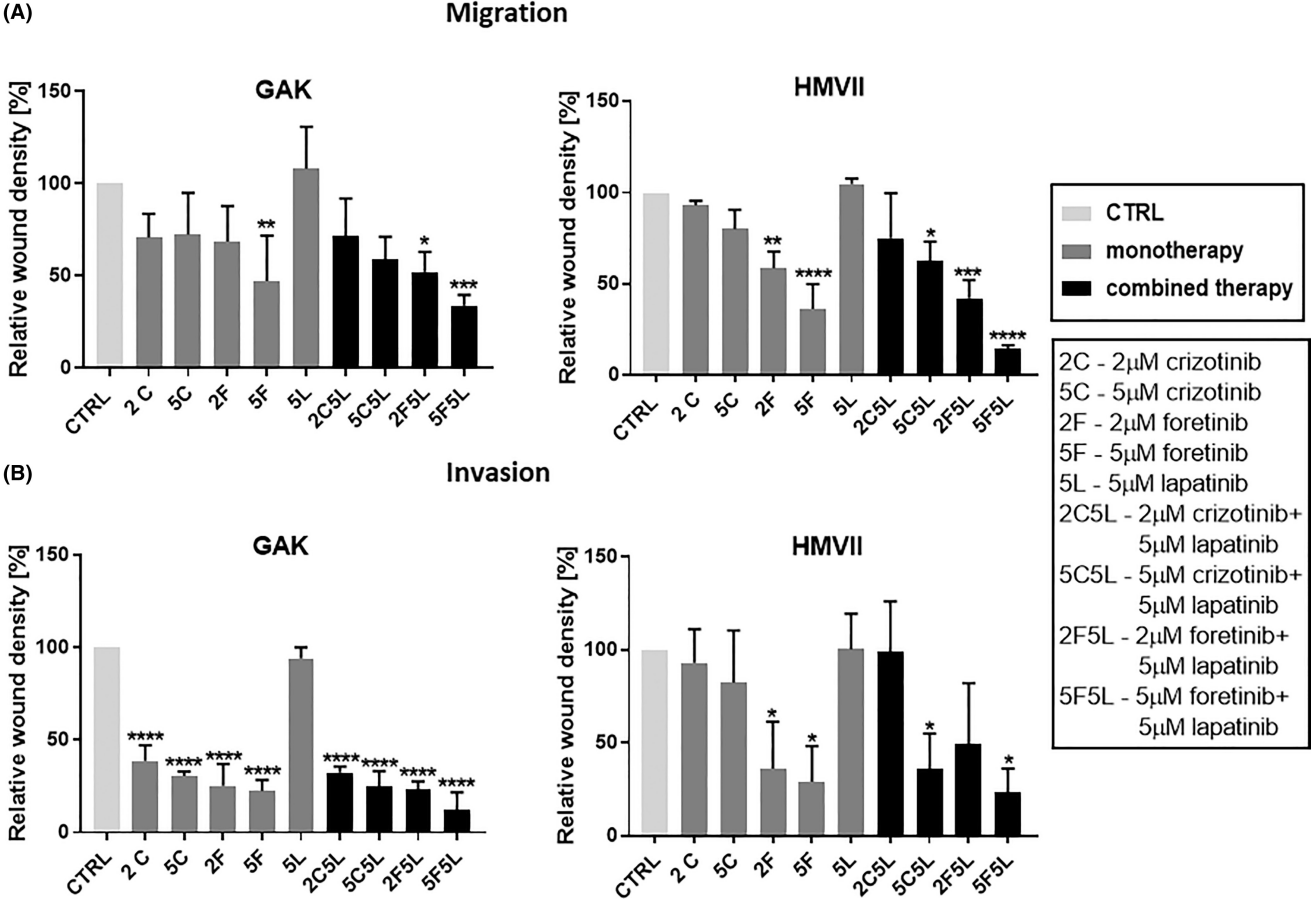
Migration and invasion abilities of mucosal melanoma cells treated with MET and EGFR inhibitors. GAK and HMVII cells were incubated with crizotinib (C), foretinib (F), lapatinib (L) or their combinations at the indicated concentrations (μM) for 48 h. Migration (A) and invasion (B) experiments on GAK and HMVII cells were conducted using wound healing method on ImageLock plates using Incucyte Live‐Cell Analysis System. Results are expressed as the mean ± SD and are based on at least three independent experiments. Migration and invasion capacity of control cells is set to 100%. For all graphs, asterisks indicate conditions statistically significant, which are different from the control. The significance level was set at *p* ≤ 0.05 (*), *p* ≤ 0.01 (**), *p* ≤ 0.001 (***) or *p* ≤ 0.0001 (****).

We also investigated the combined effect of the inhibitors crizotinib, foretinib and lapatinib on the migration and invasion capacities of MM cells. After 48 h, a significant decrease in migration capacity was observed in cells treated with combination of crizotinib/lapatinib (only for HMVII cells) or foretinib and foretinib/lapatinib (for both cell lines) in comparison with control cells (Figure 2A). The analogous results were obtained via an invasion assay in which a confluent cell population embedded between two Matrigel layers invaded formed wound (Figure 2B). The effect of inhibitors on invasion was stronger in the case of GAK cells (in this case also crizotinib and its mixes reduced cell invasive abilities), although data obtained for HMVII cells were still statistically significant.

### 3.3 Effect of EGFR and MET inhibition on downstream signalling

**FIGURE 3:**
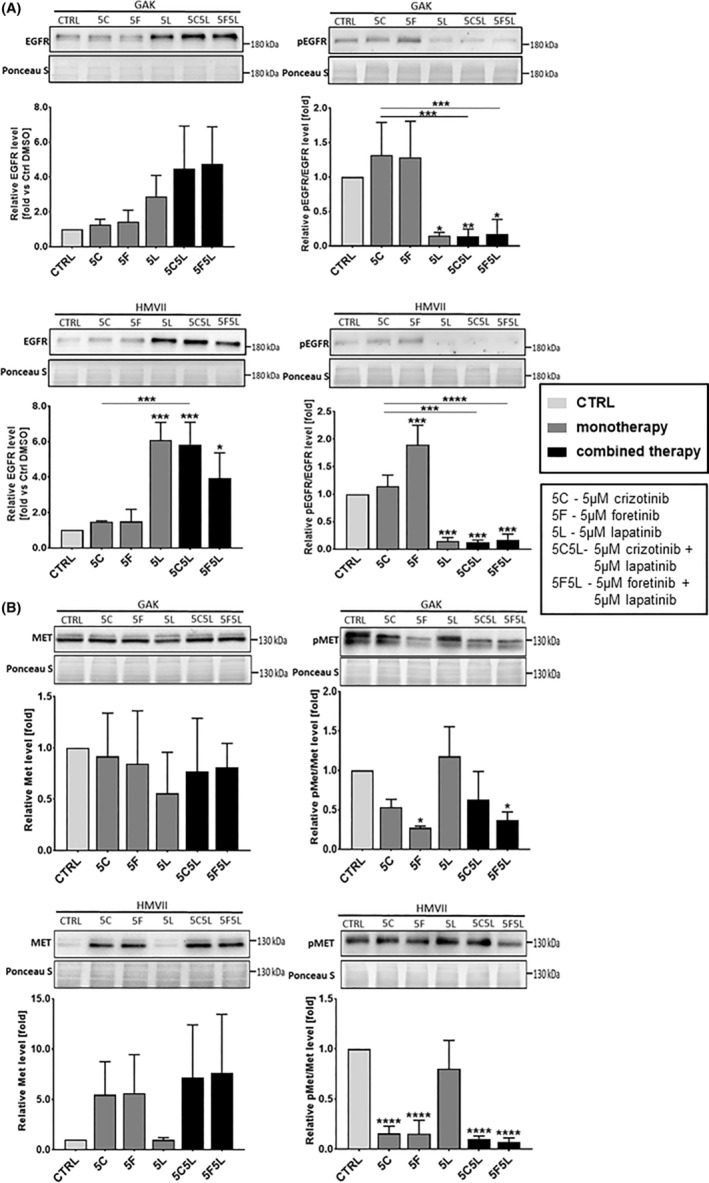
Activation of EGFR and MET receptors in mucosal melanoma cell lines treated for 24 h with inhibitors. Representative immunoblots showing pEGFR/EGFR (A) and pMET/MET (B) levels in cellular extracts of GAK and HMVII control cells and cells treated with 5 μM crizotinib (5C), 5 μM foretinib (5F), 5 μM lapatinib (5 L) or their combinations. Graphs present densitometric analysis of protein bands for EGFR, pEGFR/EGFR ratio, MET and pMET/MET ratio. The densitometric analysis for selected proteins was adjusted to the total protein content visualized in Ponceau S staining. The statistical significance was assessed versus the control. The significance level was set at *p* ≤ 0.05 (*), *p* ≤ 0.01 (**), *p* ≤ 0.001 (***) or 0.0001 ≤ (****).

**FIGURE 4:**
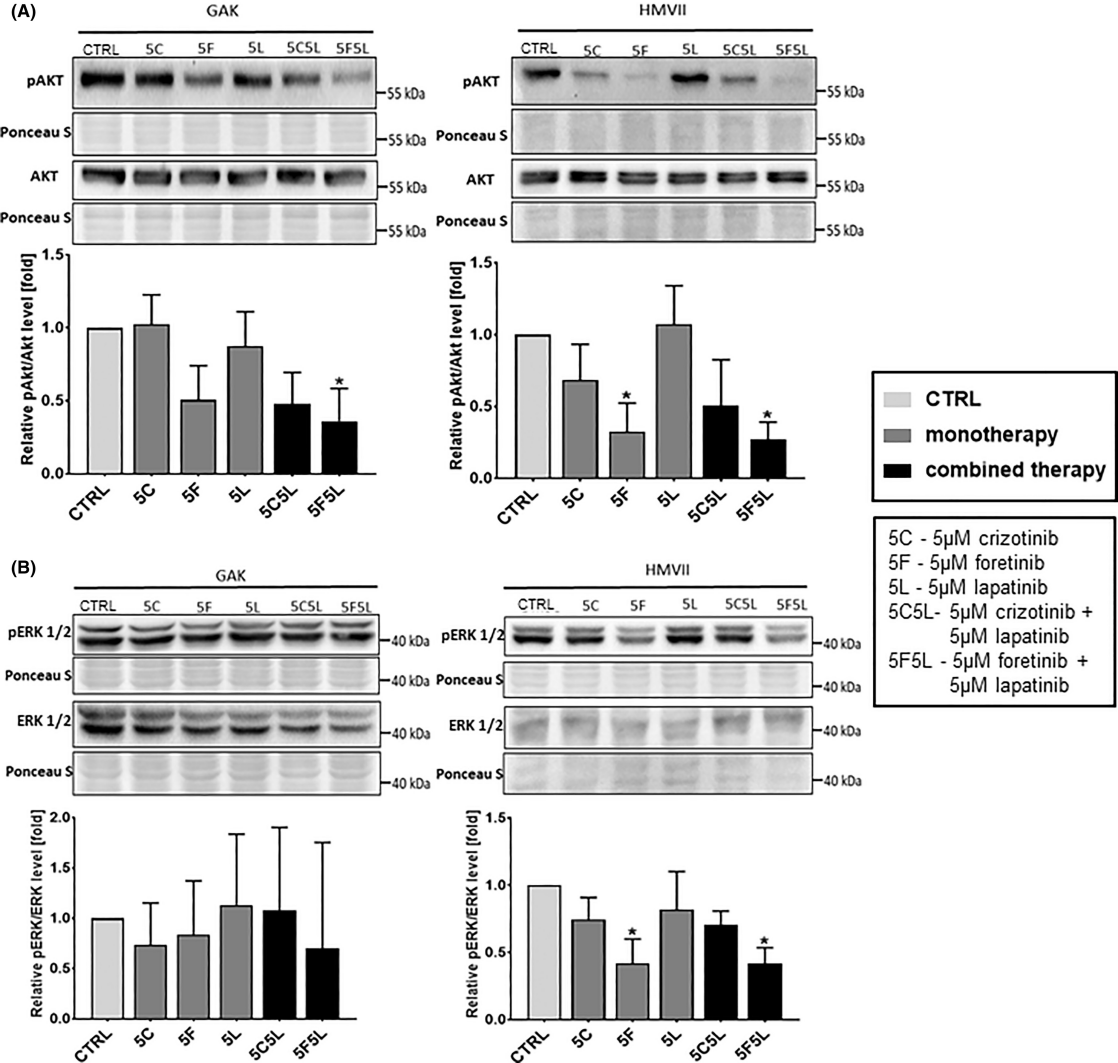
Activation of ERK1/2 and AKT kinases in mucosal melanoma cell lines treated for 24 h with inhibitors. Representative immunoblots showing pAKT/AKT (A) and pERK1/2/ERK1/2 (B) levels in cellular extracts of GAK and HMVII control cells and cells treated with 5 μM crizotinib (5C), 5 μM foretinib (5F), 5 μM lapatinib (5 L) or their combinations. Graphs present densitometric analysis of protein bands for pERK1/2/ERK1/2 ratio and pAKT/AKT ratio. The densitometric analysis for selected proteins was adjusted to the total protein content visualized in Ponceau S staining. The statistical significance was assessed versus the control. The significance level was set at *p* ≤ 0.05 (*).

Given our interest in potential crosstalk, we studied the activation state of selected proteins involved in EGFR and MET signalling pathways in GAK and HMVII cells treated with combinations of inhibitors using Western blotting analysis. Stimulation with EGF and HGF resulted in a high level of phosphorylation of the receptors, EGFR and MET, which is evident from the control samples (treated with 0.1% DMSO—drugs' solvent) shown on Figure 3. We investigated the changes in the receptor activation state (phosphorylation) and downstream signalling for both cell lines following treatment with drugs used alone or in combinations. As expected, we observed that lapatinib led to reduction of the pEGFR/EGFR ratio (Figure 3A), and crizotinib, and foretinib decreased the pMET/MET ratio in both cell lines (Figure 3B). Of interest, lapatinib used both alone and in mixtures with MET inhibitors also increased the level of the EGFR receptor in both cell lines, while foretinib and crizotinib increased the level of MET in HMVII cells. Simultaneous use of crizotinib or foretinib with lapatinib reduced the level of both phosphorylated receptors in both cell lines, except of pMET level in GAK cells treated with 5C5L combination. In selected cases, monotherapies based on these drugs were also able to exert this effect (Figure 3). In addition, we observed, that in HMVII cell line treated with foretinib as monotherapy, the pEGFR/EGFR ratio was increased, which may indicate that cancer cells compensate for the decreased pMET/MET ratio by increasing the pEGFR/EGFR ratio. Studied inhibitors also influenced EGFR and MET effectors. Combination of foretinib and lapatinib, and in the case of HMVII also foretinib used alone, decreased the pAKT/AKT ratio compared to control in both cell lines. Similar trend was observed for crizotinib and lapatinib combination, although detected reduction was not statistically significant (Figure 4A). The pERK1/2/ERK1/2 ratio was reduced only in HMVII cells treated with either foretinib or its combination with lapatinib (Figure 4B). In the case of GAK, large standard deviations resulted in no significant differences. All obtained data on the level of the described proteins in lysates from GAK and HMVII cells treated with the tested inhibitors have been collected in Table S1. These results indicate that foretinib used alone and the combinations of EGFR and MET inhibitors block the activation of pathways essential to cell growth, migration and invasion. Combined therapy of MET and EGFR inhibitors may be more effective than monotherapy, and it might help to avoid potential crosstalk of signal transduction pathways crucial for MM progression.

### 3.4 Effect of inhibitors on cells isolated from patient's biopsy

**FIGURE 5:**
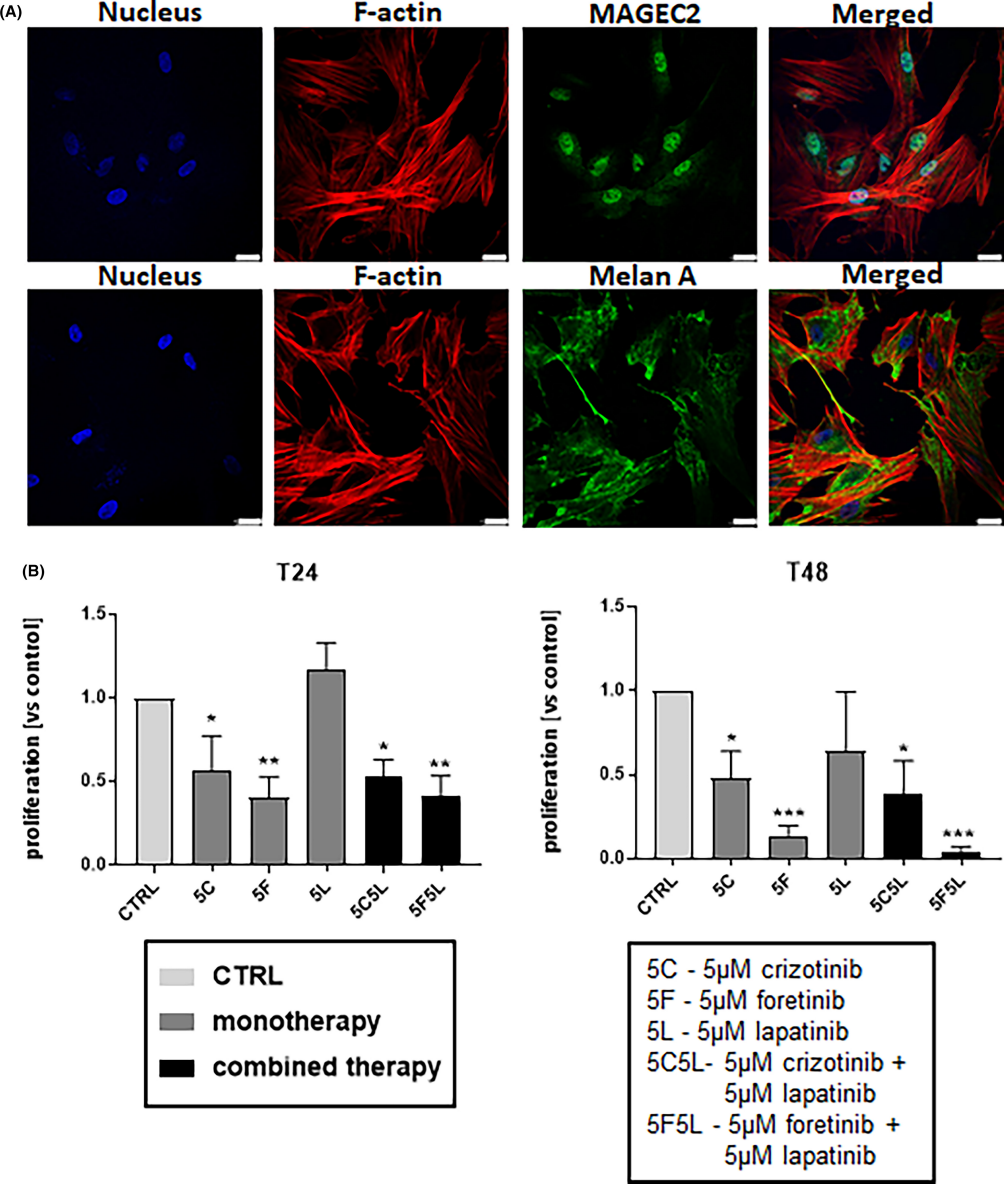
Identification of biopsy‐derived melanoma cell line JM2605 and the effect of inhibitors on its proliferation. Melanoma cells derived from the patient's biopsy were identified based on MAGEC2 and Melan A staining (A, green). Additionally, cell nuclei (A, blue) and actin cytoskeleton (A, red) were labelled in examined cells. Scale bar—25 μm. (B) Proliferation of JM2605 cells after 24 or 48 h of incubation with inhibitors—5 μM crizotinib (5C), 5 μM foretinib (5F), 5 μM lapatinib (5 L) and their mixtures were compared to those of control cells. Number of proliferating cells was calculated versus control with a control set to 1. Results expressed as the mean ± SD are based on at least three independent experiments. The statistical significance was assessed versus that of the control. The significance level was set at *p* ≤ 0.05 (*), *p* ≤ 0.01 (**) or *p* ≤ 0.001 (***).

Our next step was to evaluate the effect of crizotinib, foretinib and lapatinib separately or in combinations on MM cells isolated from a patient's vaginal melanoma. Melanoma cells were identified by MAGEC2 and Melan‐A staining (Figure 5A). The first marker recognizes tumour cells, while the second one melanoma cells.^35^, ^36^ Cells were characterized by a polygonal shape and a well‐organized cytoskeleton with a large amount of stress fibres, which is clearly visible in F‐actin staining (Figure 5A). Newly established cell line was called JM2605. Due to MM's rarity, we were able to obtain only one patient's‐derived cell line. Cells were then treated with crizotinib, foretinib and lapatinib separately or in combinations. We examined the influence of used drugs on proliferation, migration, invasion and activation of signalling pathways of these cells. We noticed that after MET inhibitors and drugs' combinations treatment cells proliferation was significantly reduced, both 24 and 48 h after drugs addition (Figure 5B).

**FIGURE 6:**
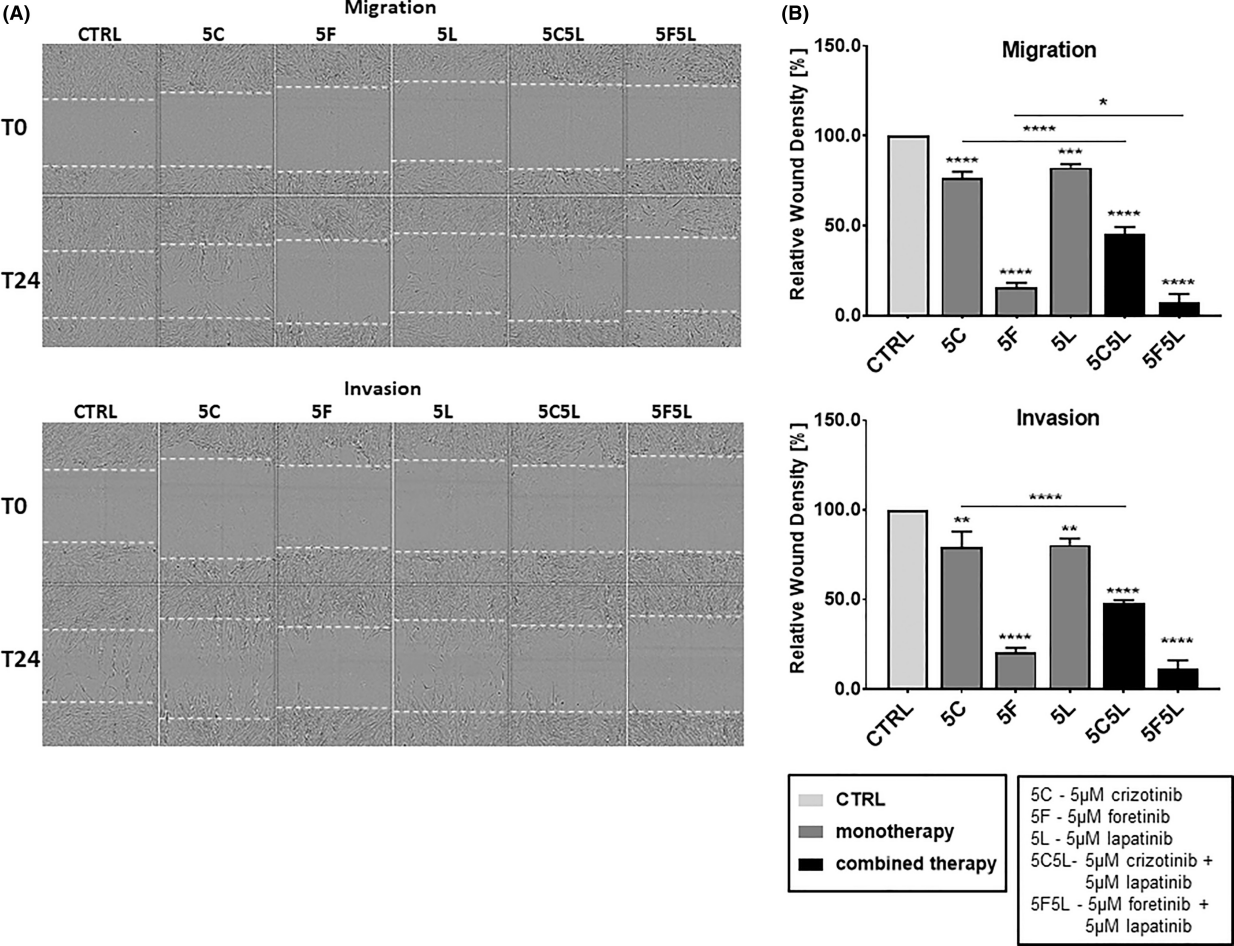
Migration and invasion capacities of biopsy‐derived melanoma cells JM2605 treated with inhibitors. Cells were seeded on a thin layer of Matrigel (migration) or embedded between two layers of Matrigel (invasion) and then incubated with 5 μM crizotinib (5C), 5 μM foretinib (5F), and 5 μM lapatinib (5 L) or their combinations at the indicated concentrations (μM) for 24 h. (A) Exemplary pictures illustrating wound closure after 24 h. (B) Relative wound density after 24 h ‐migration and invasion was continuously measured and quantified based on pictures captured with an IncuCyte® Scratch Wound Cell Migration Software Module. Results are expressed as the mean ± SD and are based on at least three independent experiments. Asterisks indicate differences between control and treated cells or between particular conditions. The significance level was set at *p* ≤ 0.05 (*), *p* ≤ 0.01 (**), *p* ≤ 0.001 (***) or *p* ≤ 0.001 (****).

Similar effect was visible when we analysed migration and invasion of examined cells (Figure 6). We noticed that all used inhibitors, applied both separately and in combinations, significantly reduced migration and invasion abilities of biopsy‐derived cells. In the case of treatment with the mixture of foretinib and lapatinib cells covered only 10% of the surface covered at the same time by control cells. We also observed that crizotinib alone was much less effective in reducing migration and invasion of examined cells than in combination with lapatinib. This effect was also visible in the case of foretinib and its mixture with lapatinib in migration test.

**FIGURE 7:**
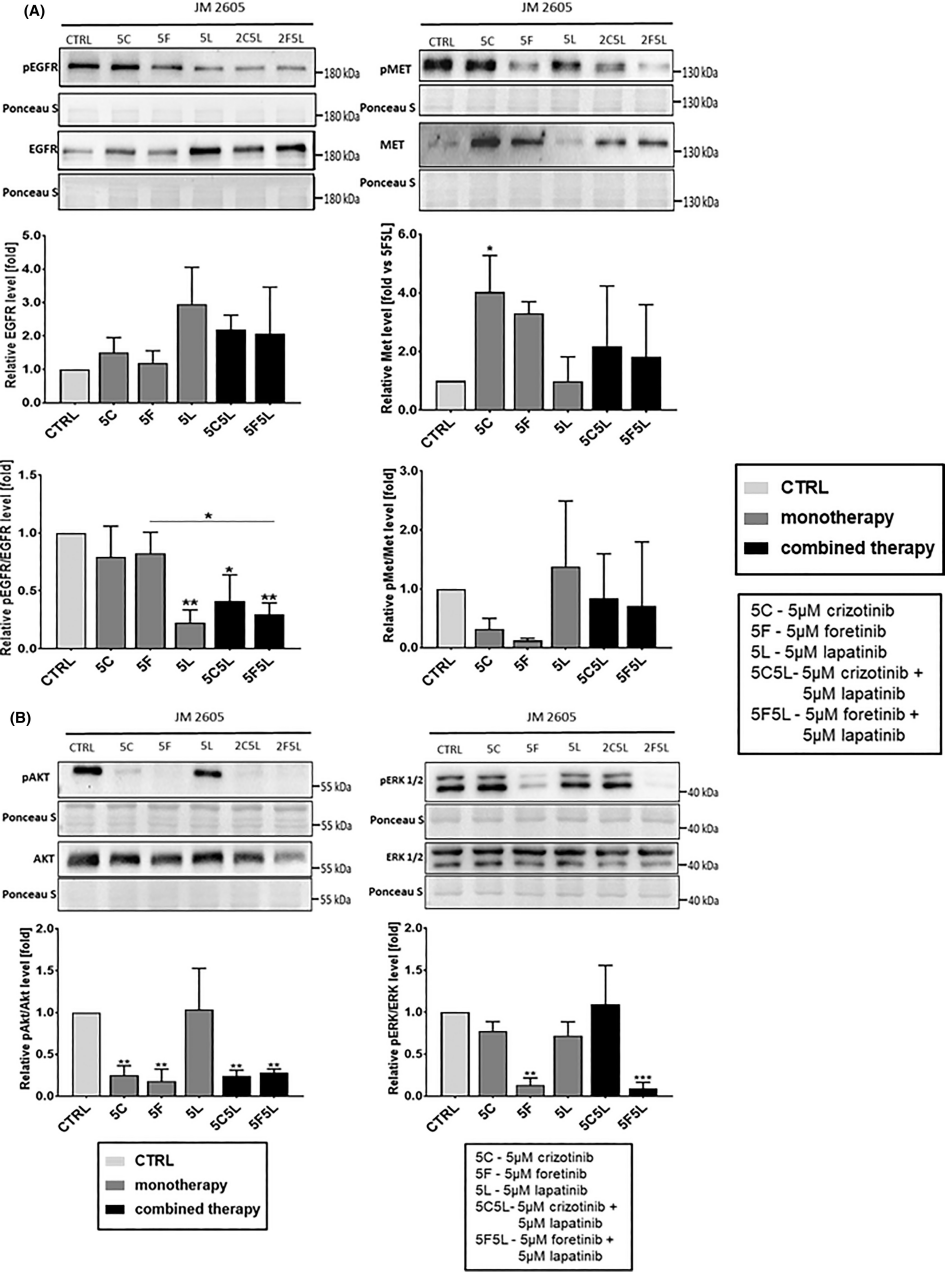
Activation of EGFR, MET, ERK1/2 and AKT proteins in biopsy‐derived JM2605 mucosal melanoma cells treated for 24 h with inhibitors. Representative immunoblots showing pEGFR, EGFR, pMET and MET (A) as well as pAKT, AKT, pERK1/2 and ERK1/2 (B) levels in cellular extracts of JM2605 control cells and cells treated with 5 μM crizotinib (5C), 5 μM foretinib (5F), 5 μM lapatinib (5 L) or their combinations. Graphs present densitometric analysis of protein bands for pEGFR/EGFR, pMET/MET, pAKT/AKT and pERK1/2/ERK1/2 ratio. The densitometric analysis for selected proteins was adjusted to the total protein content visualized in Ponceau S staining. The statistical significance was assessed versus the control. The significance level was set at *p* ≤ 0.05 (*), *p* ≤ 0.01 (**), *p* ≤ 0.001 (***).

We also examined the changes in the receptors activation state and downstream signalling after treatment with drugs, alone or in combinations. Lapatinib used as monotherapy and in combination with MET inhibitors led to reduction of pEGFR/EGFR ratio in a statistically significant manner. Crizotinib, foretinib and their combinations with lapatinib reduced pMET/MET ratio, although these differences were not statistically significant, which may be related to high SDs. Moreover, total EGFR level was increased after lapatinib and drugs' combination treatment, while total MET level was elevated after MET inhibitors treatment, although only in the case of crizotinib this difference was statistically significant (Figure 7A). The total EGFR was upregulated after lapatinib and combinations treatment also in the case of cell lines. In HMVII cell line combination of lapatinib with MET inhibitors exerted the same effect regarding total MET level (Figure 3A). Studied inhibitors influenced also EGFR and MET downstream effectors in patient‐derived cells. Crizotinib and foretinib used both alone and in combinations with lapatinib reduced the pAKT/AKT ratio compared to control (Figure 7B). In cell lines, this reduction was demonstrated only in the case of foretinib and its combination with lapatinib (Figure 4A). The pERK1/2/ERK1/2 ratio was reduced only in cells treated with foretinib and its mixture with lapatinib (Figure 7B). Similar effect was observed in HMVII cell line (Figure 4B). All obtained data on the level of the described proteins in lysates from JM2605 cells treated with the tested inhibitors have been collected in Table S1. Effectors of signalling pathways appear to be more strongly inhibited by drugs in biopsy‐derived cells than in cells representing commercially available cell lines. Of interest, the patient's vaginal melanoma harboured wildtype *BRAF*, which infers that BRAF inhibition would not be effective.

## 4 DISCUSSION

EGFR and MET, activate numerous, partly overlapping, signalling pathways that promote cell growth, invasion and cell survival with the predominant ones being RAF/ERK1/2 and PI3K/AKT pathways. Moreover, functional crosstalk of these receptors has been reported with EGFR activating MET or, conversely, with MET regulating EGFR tyrosine phosphorylation.^37^ Mueller et al.^37^ suggested that MET may influence EGFR phosphorylation through co‐association, which induces conformational changes in both receptors and regulates the kinase activity of MET. This association of EGFR and MET and their reciprocal regulation suggests that blocking activity of these kinases individually may be insufficient and indeed monotherapy based on inhibitors of these kinases frequently lead to appearance of drug resistance.^38^, ^39^ Thus, combination therapy could be a promising strategy.

Large‐scale whole genome sequencing analysis of cutaneous, mucosal and acral melanoma demonstrated frequent *MET* aberrations.^40^, ^41^ Moreover, EGFR expression in melanomas was reported to influence the sentinel lymph node metastasis and *EGFR* amplification was related to the weak response of patients with mucosal and acral melanoma to immune checkpoint inhibitors based therapy.^25^, ^42^, ^43^ It has been also shown that *MET* and *EGFR* overexpression, amplification of gene copies or mutations are associated with poor prognosis for the patients.^44^, ^45^ Treatment of the EGFR‐expressing melanoma cell line with anti‐EGFR antibody—cetuximab reduced the invasive capacity of the cells, but did not alter cell viability or growth,^43^ while cabozantinib, an inhibitor of tyrosine kinases including MET, VEGF (vascular endothelial growth factor) and AXL (tyrosine‐protein kinase receptor UFO), led to longer progression‐free survival and reduction in tumour size in the majority of patients with uveal, cutaneous and mucosal melanoma.^46^, ^47^ The findings of Cao et al.^48^ suggested that crizotinib, which is also an inhibitor of ROS‐1 (ROS protooncogene 1, receptor tyrosine kinase), can be an option to improve overall survival and quality of life of patients with metastatic ROS1‐fusion MM. It was previously demonstrated that in non‐small cell lung cancer cells, MET overexpression upregulated EGFR downstream signalling and the use of both EGFR/HER2 and MET tyrosine kinase inhibitors led to a maximal growth inhibition.^49^ Kim et al.^50^ also indicated that the MET inhibitor PHA‐665752 showed a synergistic effect in combination with an EGFR inhibitor, erlotinib, in the triple negative breast cancer cell line, MDA‐MB‐468. In another study, Liu et al.^51^ successfully applied foretinib in combination with lapatinib against various cancer cell lines, but melanoma cells were not tested. Cytotoxic effect of above‐mentioned drugs was also confirmed by us in studies of triple negative breast cancer and CM.^23^, ^52^

In this study, we showed that combinations of selected MET and EGFR inhibitors, crizotinib, foretinib and lapatinib, respectively, act effectively in the two commercially available MM cell lines as well as MM cells isolated from patient's vaginal melanoma. Continuous stimulation with EGF and HGF leads to ligand‐based activation of signalling pathways as is occurring in the tumour. The inhibitors act synergistically to reduce proliferation of examined cells. Moreover, the simultaneous usage of inhibitors of these two receptors might prevent crosstalk between them. Applied combinations of MET and EGFR inhibitors also reduced AKT and ERK1/2 activation as well as cell migration and invasion, which is crucial functional feature to form regional and distant metastases. In detail, we observed that the pairs of drugs inhibited cell proliferation, though the combination of 2 μM crizotinib and 7.5 μM lapatinib was significantly more effective in cell lines than only 2 μM crizotinib, while 2 μM foretinib and 7.5 μM lapatinib was significantly more effective in cell lines than only 2 μM foretinib. This demonstrates the validity of using a combination of inhibitors instead of monotherapy, especially that both drugs' pairs exert synergistic effect on proliferation of examined cells. Moreover, used drugs were able to reduce MM migration and invasion usually more effectively than monotherapy. To better mimic the microenvironment of migrating cells, we investigated MM cell motility both in conditions imitating the migration on the surface of basement membrane, and in conditions, reflecting invasion through the surrounding tissues. Tested drugs led to stronger inhibition of invasion in the case of GAK cells. The observed effect may be related to the fact that *BRAF* gene is not mutated in GAK cells, whereas in HMVII cells in gene encoding BRAF is present mutation that leads to Gly469Val substitution. As a result of the mutation, the kinase becomes overactive, stimulates cell migration and invasion more strongly, and probably also makes cells less sensitive to EGFR and MET inhibitors.

Our results are in line with data obtained by Lee et al.^53^ who noticed that ME22S (a novel EGFR/MET bispecific antibody) remarkably reduced HGF‐stimulated migration and invasion of laryngeal carcinoma cells. Moreover, Xu et al.^54^ showed that pair of EGFR and MET inhibitors decreased the rate of wound closure and invasion of head and neck carcinoma cells. Das et al.^55^ also indicated that combination of afatinib (ERBB family inhibitor) and crizotinib (MET inhibitor) had a synergistic effect, promoting cell death of CM. It downregulated also migratory and invasive capacity of examined cells independently of their *BRAF*/*NRAS* mutational status. Furthermore, the combinations of inhibitors attenuated tumour growth rate and induced DNA damage in vivo. It is worth noting that this form of therapy had minimal therapy‐related toxicity in mice. Our study also showed that the level of EGFR was elevated in HMVII cell line after lapatinib monotherapy and its combinations with crizotinib or foretinib. MET level was also increased in HMVII cells after MET inhibitors treatment. We speculate that the observed phenomenon may be due to the compensatory effect—cells in which inhibitors have lowered phosphorylated EGFR or MET levels try to increase the amount of these proteins by elevating EGFR/MET expression. pEGFR/EGFR ratio was reduced in both cell lines treated with lapatinib and its combinations with MET inhibitors, while pMET/MET ratio was downregulated in cells treated with MET inhibitors and their combinations with lapatinib. We also noticed that in cell lines treated singly with either crizotinib or foretinib, the pEGFR/EGFR ratio was elevated, which may indicate that the cancer cells compensate for the reduced pMET/MET ratio by increasing the pEGFR/EGFR ratio. This may justify the proposed combination of EGFR and MET inhibitors in therapy. Moreover, the level of main effectors of EGFR/MET, which are mediators of the pro‐survival/pro‐migratory signalling, was examined. Under the influence of foretinib and both combinations of inhibitors pAKT level was decreased in both cell lines, while pERK1/2 level was reduced only in HMVII in statistically significant manner. It is worth noting that the applied therapy was effective against the tested lines, regardless of the mutational status of *BRAF*.

We observed a similar tendency in response to treatment in patient's tumour‐derived cells compared to the cell lines. Their proliferation, migration and invasion as well as activation of effectors of signalling pathways (ERK1/2, AKT) were substantially decreased after treatment with MET inhibitors and their combinations with lapatinib. The only exception was ERK1/2 activation, which was reduced only under the influence of 5F and 5F5L. We also observed that the mixture of crizotinib and lapatinib significantly reduced the migration and invasion of cells compared to corresponding monotherapy. It is also visible in the case of foretinib and lapatinib, but in terms of invasion it is not statistically significant. The above‐mentioned data strengthen the hypothesis about the need to use inhibitors of both receptors in combination. For patients with wildtype *BRAF*, anti‐EGFR/anti‐MET therapy could be a promising therapeutic option.

In summary, our preliminary study highlights the possibility for future in vivo and clinical verification of the utility of MET and EGFR inhibitors as a potential therapeutic strategies in mucosal melanoma. Importantly, we confirmed the results obtained on commercially available cell lines on tumoral cells derived from a patient's vaginal melanoma. Moreover, our studies support the findings that targeting multiple RTKs might block signalling crosstalk and prevent appearance of resistance to kinase inhibitors in mucosal melanoma cells.

## AUTHOR CONTRIBUTIONS

**Aleksandra Simiczyjew:** Conceptualization (equal); data curation (equal); formal analysis (equal); investigation (equal); methodology (equal); validation (equal); visualization (equal); writing – original draft (equal); writing – review and editing (equal). **Justyna Wądzyńska:** Formal analysis (equal); investigation (equal); methodology (equal); writing – review and editing (equal). **Magdalena Kot:** Investigation (equal); methodology (equal); writing – review and editing (equal). **Marcin Ziętek:** Methodology (equal); writing – review and editing (equal). **Rafał Matkowski:** Methodology (equal); writing – review and editing (equal). **Mai Hoang:** Conceptualization (supporting); writing – review and editing (equal). **Piotr Donizy:** Conceptualization (equal); funding acquisition (equal); supervision (supporting); writing – review and editing (equal). **Dorota Nowak:** Conceptualization (equal); funding acquisition (equal); project administration (equal); supervision (lead); writing – review and editing (equal).

## CONFLICT OF INTEREST STATEMENT

The authors declare that they have no competing interests.

## Supporting information


Table S1.

## Data Availability

The data that support the findings of this study are available from the corresponding author upon reasonable request.
