# Supplementary material for: Combinations of EGFR and MET inhibitors reduce proliferation and invasiveness of mucosal melanoma cells
Source: J Cell Mol Med. 2023 Sep 7;27(19):2995–3008. doi: 10.1111/jcmm.17935 (PMC10538264; doi:10.1111/jcmm.17935)
Supplement: Supplementary file 1 — Table S1. [file JCMM-27-2995-s001.docx]

**Supplementary Table 1 The level of selected proteins in cells treated with inhibitors, expressed as a percentage in relation to the control**

| **GAK** | **CTRL** | **5C** | **5F** | **5L** | **5C5L** | **5F5L** |
| --- | --- | --- | --- | --- | --- | --- |
| **pEGFR** | 100 | 139,722 | 145,097 | 37,9287 | 39,28229 | 40,6806 |
| **EGFR** | 100 | 78,3584 | 81,0013 | 358,034 | 433,7752 | 539,4168 |
| **pMET** | 100 | 59,1004 | 29,8685 | 77,7908 | 47,587 | 34,10573 |
| **MET** | 100 | 91,6813 | 84,6431 | 55,6483 | 77,14415 | 80,88973 |
| **pERK 1/2** | 100 | 78,5395 | 74,0644 | 95,9763 | 78,55973 | 53,335 |
| **ERK 1/2** | 100 | 134,908 | 150,529 | 143,601 | 151,4154 | 290,2476 |
| **pAKT** | 100 | 88,5688 | 45,2049 | 71,3974 | 44,85813 | 40,6183 |
| **AKT** | 100 | 89,862 | 85,228 | 76,8515 | 88,97337 | 138,4247 |
|  |  |  |  |  |  |  |
| **HMVII** | **CTRL** | **5C** | **5F** | **5L** | **5C5L** | **5F5L** |
| **pEGFR** | 100 | 155,354 | 253,404 | 96,568 | 68,08565 | 56,85909 |
| **EGFR** | 100 | 90,3708 | 97,798 | 350,701 | 253,0293 | 239,7546 |
| **pMET** | 100 | 65,6128 | 65,3025 | 93,557 | 76,16023 | 49,59107 |
| **MET** | 100 | 546,948 | 562,865 | 97,7147 | 716,4018 | 766,2573 |
| **pERK 1/2** | 100 | 93,8176 | 53,7224 | 97,6236 | 83,75318 | 39,8352 |
| **ERK 1/2** | 100 | 128,906 | 130,844 | 109,917 | 104,247 | 93,29605 |
| **pAKT** | 100 | 70,004 | 41,7337 | 97,9773 | 58,56225 | 39,96433 |
| **AKT** | 100 | 88,1528 | 89,6559 | 90,2267 | 104,2149 | 123,5361 |
|  |  |  |  |  |  |  |
| **JM2605** | **CTRL** | **5C** | **5F** | **5L** | **5C5L** | **5F5L** |
| **pEGFR** | 100 | 112,296 | 101,911 | 73,4576 | 90,2881 | 52,05167 |
| **EGFR** | 100 | 145,369 | 88,1914 | 227,261 | 151,0899 | 115,1988 |
| **pMET** | 100 | 117,933 | 40,833 | 82,731 | 87,28461 | 17,9861 |
| **MET** | 100 | 403,68 | 330,398 | 99,6081 | 218,9796 | 181,6704 |
| **pERK 1/2** | 100 | 83,5972 | 13,7658 | 58,7833 | 81,57815 | 6,428097 |
| **ERK 1/2** | 100 | 106,423 | 98,9931 | 81,7166 | 75,24933 | 68,06176 |
| **pAKT** | 100 | 22,2189 | 13,4192 | 85,1446 | 17,29513 | 16,7277 |
| **AKT** | 100 | 87,6905 | 75,9597 | 87,4899 | 78,7783 | 60,85867 |

*Abbreviations: CTRL – control, 5C - 5µM crizotinib, 5F - 5µM foretinib, 5L - 5µM lapatinib, 5C5L - 5µM crizotinib +5µM lapatinib, 5F5L - 5µM foretinib + 5µM lapatinib, EGFR – epidermal growth factor receptor, pEGFR – phosphorylated EGFR, MET – hepatocyte growth factor receptor, pMET – phosphorylated MET, ERK1/2 - extracellular signal-regulated kinase, pERK1/2 – phosphorylated ERK1/2, AKT - Protein kinase B, pAKT – phosphorylated AKT*
